# Complete chloroplast genome of *Lycoris sprengeri* (Amaryllidaceae) and genetic comparison

**DOI:** 10.1080/23802359.2019.1676673

**Published:** 2019-10-15

**Authors:** Fengjiao Zhang, Weibing Zhuang, Xiaochun Shu, Tao Wang, Zhong Wang

**Affiliations:** Jiangsu Key Laboratory for the Research and Utilization of Plant Resources, Institute of Botany, Jiangsu Province and Chinese Academy of Sciences (Nanjing Botanical Garden Mem. Sun Yat-Sen), Nanjing, PR China

**Keywords:** *Lycoris sprengeri*, complete chloroplast genome, genetic comparison

## Abstract

*Lycoris sprengeri* is native to China and has various variations. It belongs to the Amaryllidaceae family, which contains abundant alkaloids for medical use and also was planted as garden bulbous flowers. In this study, we assembled the complete chloroplast (cp) genome of *L. sprengeri* by DNA sequencing, which will improve the complete cp genomic information for analysis of phylogenetic relationships and germplasm identification in *Lycoris*. The whole cp genome is 158,687 bp, which contained a large single-copy region (LSC) of 86,489 bp, a small single-copy region (SSC) of 18,540 bp, and a pair of inverted repeats (IRs) of 26,829 bp. A total of 137 genes were annotated, including 87 protein coding genes (PCGs), 42 tRNA, and 8 rRNA genes. Phylogenetic tree analysis revealed that the close relationship of three species of *Lycoris* (*L. sprengeri*, *L. radiate*, and *L. squamigera*) in the Amaryllidaceae family.

*Lycoris sprengeri* is native to central China, and a member of the Amaryllidaceae family with abundant alkaloids for medical use (Wu et al. 2014) and beautiful flower for bulbous flowers. However, because of the high-frequency interspecific hybridization in *Lycoris*, there are various variants that co-existed in one species (Zhang et al. 1999). Although some researchers have tried to clarify their interspecific relationships and hybrid origin of *Lycoris* species by ITS and rDNA sequences (Shi et al. 2006; Quan et al. 2012), the evidence was not enough to prove it. In the Genbank, limited chloroplast (cp) genome fractions of *L. sprengeri* can be found, which are insufficient to the phylogenetic analysis. Here, we assembled the complete cp of *L. sprengeri*, described the characteristics and constructed the phylogeny tree for genetic comparison. The results will provide more evidence for the phylogeny analysis and germplasm identification of *Lycoris* species.

*L. sprengeri* bulbs were planted in Nanjing Botanical Garden, Mem. Sun Yat-sen (E118_83, N32_06), Nanjing, China. Specimens (no. NAS00585503) were stored at herbarium of Institute of Botany, Jiangsu Province and Chinese Academy of Science. Fresh leaves were collected for DNA extraction according to the instruction of plant DNA isolation reagent (Code: D9194, TaKaRa, Beijing, China). DNA purity and integrity were detected using a NanoDrop spectrophotometer and agarose gel electrophoresis, then qualified DNA was used for library construction. The DNA sequencing was conducted by Illumina Noveseq at Novogene company (http://www.novogene.com/). As a result, 172 million reads (paired-end 150) were generated and 10 million reads were assembled to *L. sprengeri* cp genome, with 10,352-fold organelle coverage. NOVOPlasty version 2.6.2 (https://github.com/ndierckx/NOVOPlasty) (Dierckxsens et al. 2017) was used as an assembler and published *L. squamigera* cp genome (GenBank accession MH118290.1) (Jin et al. 2018) was chosen as a reference. Web server CPGAVAS2 (http://www.herbalgenomics.org/cpgavas2) (Shi et al. 2019) was performed to genome annotation, visualization, and tandem repeats identification.

The cp genome of *L. sprengeri* was deposited in GenBank (accession no. MN158986). Whole cp genome was 158,687 bp with 37.8% GC content, which comprised a large single-copy (LSC) region of 86,489 bp, a small single-copy (SSC) region of 18,540 bp and two equal lengths inverted repeat (IR) regions of 26,829 bp. A total of 137 genes were predicted, which contained 87 protein-coding genes (PCGs), 42 tRNA, and 8 rRNA genes. In PCGs, 16 splitting genes contained one intron and two genes (*ycf3* and *clpP*) contained two introns, which is consistent with the *L. radiata* cp genome (Zhang et al. 2019, accepted).

Phylogenetic analysis was completed using the whole cp genome of 17 species belonged to five related families. The CLUSTAL format was aligned by MAFFT version 7.427 with default parameters (https://mafft.cbrc.jp/alignment/server/) (Rozewicki et al. 2019), then the phylogenetic tree was constructed by neighbour-joining (NJ) with 1000 bootstrap replicates (Figure 1). From the phylogenetic tree, we could see that all species were clustered on the correct clades of family, *L. sprengeri* was grouped together with *L. Squamigera*, and *L. radiata* in *Lycoris*, together with other seven species in the Amaryllidaceae family.

**Figure 1.**
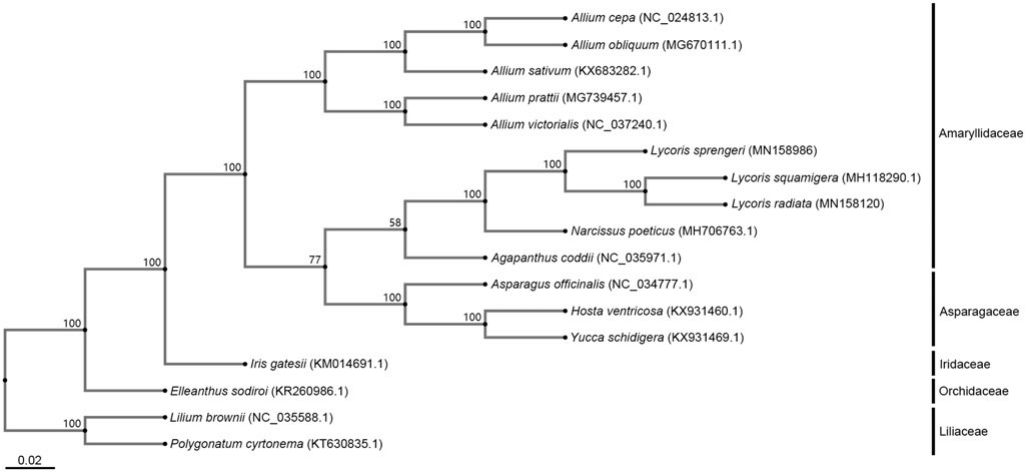
Phylogenetic tree analysis using the cp genome sequences of 17 species in five families, which revealed the close relationship among *L. sprengeri*, *L. radiata,* and *L. squamigera*. Numbers on the nodes were the bootstrap value from 1000 replicates. Genbank accession number of each species was presented in the brackets after species’ names.
